# Preparation of glutathione loaded nanoemulsions and testing of hepatoprotective activity on THLE-2 cells

**DOI:** 10.3906/kim-2007-54

**Published:** 2021-04-28

**Authors:** Ozan YEŞİLTEPE, Emine GÜLER ÇELİK, Caner GEYİK, Zinar Pınar GÜMÜŞ, Dilek ODACI DEMİRKOL, Hakan COŞKUNOL, Suna TİMUR

**Affiliations:** 1 Institute on Drug Abuse Toxicology and Pharmaceutical Science, Ege University, İzmir Turkey; 2 Department of Biochemistry, Faculty of Science, Ege University, İzmir Turkey; 3 Department of Medical Biochemistry, Faculty of Medicine, İstinye University, İstanbul Turkey; 4 Central Research Testing and Analysis Laboratory Research and Application Center, Ege University, İzmir Turkey; 5 Department of Psychiatry, Faculty of Medicine, Ege University, İzmir Turkey

**Keywords:** Ethanol, *Nigella sativa*, glutathione, nanoemulsion, hepatoprotective activity

## Abstract

To improve bioavailability and stability of hydrophobic and hydrophilic compounds, nanoemulsions are good alternatives as delivery systems because of their nontoxic and nonirritant nature. Glutathione (GSH) suffers from low stability in water, where its encapsulation in nanoemulsions is a powerful strategy to its stability in aqueous systems. The aim of this study was to obtain nanoemulsions from the hydrophobic/hydrophilic contents of *N. sativa* seed oil so as to improve GSH stability along with bioavailability of *N. sativa* seed oil. Then, the prepared nanoemulsions were tested for in vitro hepatoprotective activity against ethanol toxicity. To the best of our knowledge, there is no study on the test of nanoemulsions by the combination of *Nigella sativa* seed oils and GSH in hepatoprotective activity. Here, nanoemulsions with different contents were prepared using *Nigella sativa* seed oils. Content analyses and characterisation studies of prepared nanoemulsions were carried out. In order to investigate the protective effects against to ethanol exposure, THLE-2 cells were pretreated with nanoemulsions for 2 h with the maximum benign dose (0.5 mg/mL of nanoemulsions). Ethanol (400 mM) was introduced to pretreated cells and nontreated cells for 48- or 72-h periods, followed by cell viability assay was carried out. Fluorescence microscopy tests revealed the introduction of the nanoemulsions into THLE-2 cells. The findings show that nanoformulations have promising in vitro hepatoprotective effects on the THLE-2 cell line against ethanol exposure.

## 1. Introduction

Therapeutic effects of various compounds are limited by their low solubility and stability in water. Encapsulation of therapeutic molecules in delivery systems increases their bioavailability and stability along with guiding their transportation to the target sides within body or cells [1]. Among the encapsulation approaches, vesicular architectures such as liposomes (FDA-approved), micelles or nanoemulsions have great attention because of their advantages such as lower cytotoxicity and minimum to no side effects. Nanoemulsions, an easy way to prepare nanocarriers, are prepared as oil/water or water/oil dispersions using surfactants as stabilizing agents, where sonication is used to obtain the nanoemulsions with desired size [2,3]. 

It is known that many of plants and their seeds are used in traditional medicine. Especially in recent years, the studies on the use of seed oils for food additives and therapeutic purposes have gained momentum. In this direction, seed oils are utilized for their natural properties such as hepatoprotective activity, antioxidant activity and wound healing. This is because a variety of plant species and their seeds have been tested in vitro on various cell lines [4–6]. *Nigella sativa*, belonging to Ranunculaceae plant family, is used for the prevention and treatment of many diseases worldwide [7,8]. *N. sativa* seed oil is known for its high antioxidant, antiinflammatory, antitumor, antimicrobial and analgesic activities as presented in literature [7–10]. It has rich content of polyphenols including phenolic acids and flavonoids [11], but low solubility of *N. sativa* oil content limits its usage as therapeutic agent. Therefore, it is necessary to prepare a safety formulation for carrying its hydrophobic contents. In previous studies, nanoemulsion formulations were prepared with *N. sativa* seed oil along with enriching with various chemicals, including herbal extracts and phytochemicals. In previous studies, the efficacy of these formulations were tested on various cell lines in vitro [4,6]. In this study, glutathione (GSH) was used for enrichment of *N. sativa* oil nanoemulsions, which is a tripeptide composed of glutamine, cysteine and glycine. GSH takes an active role in defence system, nutrient metabolism and other cellular events [12,13]. The absence of GSH causes oxidative stress. In addition, it is widely used as pharmaceutical, food additive and also in the cosmetic industry [14]. In literature, there are some clinical studies with low power suggest that GSH might ameliorate the effects of alcohol [15,16]. Because of the low stability of GSH in water, loading of GSH in delivery systems significantly increases its stability, for instance loading GSH in nanoemulsions using liquid paraffin-oil and surfactants increased the stability of GSH in aqueous conditions [17]. 

Ethanol induces oxidative stress through induction of ROS production, whose negative impact is lethal to liver tissue. Among the approaches, consumption of antioxidant compounds or undergoing an antioxidant therapy is demonstrated as a strong way of dealing with ethanol toxicity that effects liver [18–20]. Antioxidants have a major role in scavenging of free radicals, which are abundant in cell [20–23]. Due to these effects, *N. sativa* seed oil was chosen to prepare nanoformulations in our study. 

The aim of this study was to obtain nanocarriers for hydrophobic/hydrophilic compounds of *N. sativa* seed oil along with improving the stability of GSH. Reduced levels of GSH cause oxidative stress. Alcohol consumption, even at lower concentrations, causes the production of ROS and DNA damage [24]. *N. sativa* seed oil nanoemulsions, enriched with GSH by different formulations and analysed using HPLC, was used in order to prevent negative effects caused by alcohol. The obtained nanoformulation, containing *N. sativa* seed oil and GSH, was tested to eliminate the damage of liver cells caused by alcohol exposure. The characterized nanoemulsions were applied to THLE-2 liver cell line, and their protective performance was tested against ethanol exposure by using in vitro studies, where hepatoprotective activity, cytotoxicity and imaging of cells were performed [25].ATTC (2016). THLE-2 ATCC CRL-2706TM Homo sapiens liver/left lobe [online]. Website: https://www.lgcstandards-atcc.org/products/all/CRL-2706.aspx?geo_country=tr# [accessed 18 02 2017]. To the best of our knowledge, for the first time, an alcohol model was established with THLE-2 cells and the most effective formulations were identified in accordance with the results obtained. To the best of our knowledge, there is no study about protective and antioxidant effects of GSH including nanoemulsions on liver cells. 

## 2. Materials and methods

### 2.1. Materials

*N. sativa* oil, extracted by using supercritical CO_2_ technique, purchased from local pharmacy (İzmir, Turkey). L-α-phosphatidylcholine, Tween 80, glutathione (GSH), thymoquinone (TQ), methanol, ethanol, hexane, methyl tert-butyl ether, 2-propanol were obtained from Sigma-Aldrich (St. Louis, MO, USA). Folin–Ciocalteu reagent, gallic acid (3,4,5-trihydroxybenzoic acid), 3-(4,5-dimethylthiazolyl-2)-2,5- diphenyltetrazolium bromide (MTT), sodium dodecyl sulfate (SDS), hydrochloric acid (HCl), diamino-2-phenylindol (DAPI), epidermal growth factor (EGF), phosphoethanolamine and were purchased from Sigma-Aldrich.

Bronchial Epithelial Cell Growth Medium (BEGM) bullet kit, penicillin/streptomycin (10,000 UI/mL), L-glutamine (200 mM) trypsin/EDTA (0.05% Trypsin; 0.20 g/L EDTA), and phosphate buffered saline (PBS) used in cell culture experiments were obtained from Lonza (Verviers, Belgium). Fetal bovine serum (FBS) was purchased from Biowest (Riverside, MO, USA). 

### 2.2. Preparation of the nanoemulsions

The nanoformulations were prepared as described in our previous studies [4,6]. The aim of preparation of the nanoemulsion formulations was to enhance bioavailability of the hydrophobic compounds of the seed oil, which are not soluble except organic solvents such as DMSO. This limits their usability because of that fact that organic solvents pose toxicity to living organisms. There were two different *N. sativa* nanoemulsions, where both formulated as oil in water (O/W) 20:80. The oil phase of the nanoemulsions included 3.0% (w/w) Tween 80 as emulsifier and 3.0% (w/w) while lecithin was used as emulsifier for aqueous phase [4,6]. Additionally, A1 formulation included 1.0 mM GSH. Table 1 shows the constituents of aqueous and oil phases of the nanoemulsions. Both aqueous and oil phases of the emulsions were stirred using ultrasonic processor (Vibra cell, Sonics & Materials, Inc., Newtown, CT, USA) for 10 min (30 s pulse) with amplitude of 40%. Sonication process was performed in ice bath to avoid excess heating. The *N. sativa* seed oil nanoemulsions were stored at +4 °C under dark conditions. 

**Table 1:** Constitution of hydrophobic and hydrophilic phases of prepared nanoemulsions.

Component	Formulations	Aqueous phase			Oil phase	
		Distilled water	GSH	Tween 80	N. sativa oil	Lecithin
w/w (%)	A1	-	80	a	20	a
	A2	80	-	a	20	a

a Final concentrations of emulsifying agents, Tween 80 and lecithin, were 3.0% (w/w).

### 2.3. Particle size

Dynamic light scattering (DLS, Zetasizer Nano ZS, Malvern Panalytical, Malvern, UK) was used to determine the size, polydispersity index (PDI) and zeta potential of the BSO nanoemulsions in triplicates.

### 2.4. Determination of total phenolic contents in the nanoemulsions

Total phenolic contents of the *N. sativa* nanoemulsions were evaluated spectrophotometrically using Folin–Ciocalteu reagent [4,26,27]. Briefly, 200 µL of 0.2 N Folin–Ciocalteu reagent (0.2 N) and 40 µL of nanoemulsion were mixed. Sodium carbonate (20%) was added to the mixture and incubated for 2 h at room temperature. After the incubation period, absorbance of the mixtures was measured at 765 nm (Thermo Fischer EVO 60, Madison, WI, USA). The total phenolic content of nanoemulsions was calculated with the help of gallic acid calibration curve as gram gallic acid equivalents per mg of nanoemulsion.

### 2.5. DPPH radical scavenging activity

The radical scavenging activity of the nanoemulsions was carried out using the colorimetric DPPH assay, which is based on reduction of 2,2-diphenyl-1-picrylhydrazyl (DPPH) [4,5,26,28]. Nanoemulsions with different concentrations were treated with DPPH (0.2 mM) for 30 min. Then, the absorbance of samples was measured at 517 nm (Thermo Fischer EVO 60). The percentage radical scavenging activities of the nanoemulsion samples were calculated by comparing nanoformulations. Experiments were carried out in triplicates.

### 2.6. Chromatographic analysis

Chromatographic separation and analysis of GSH were carried out with Agilent 1260 Infinity HPLC instrument (equipped with multiwavelength detector - MWD) using a 4.6 × 150 mm Eclipse XDB-C18 column (5.0 μm particle size). The mobile phase consisting of water and acetonitrile (75:25, v:v) and the analyses were performed isocritically at 1.0 mL/min, 210 nm and 40 °C. Twenty microliters injection volumes were applied for all the standards and samples.

Analyses were carried out according to studies of Gumus et al. and Guler et al. [4,6]. In brief, the amount of TQ contained in the *N. sativa* seed oil was determined by Agilent 1100 HPLC instrument (equipped with gradient pump and DAD detector) with using 4.6 × 150 mm Agilent Eclipse XDB-C18 column (5.0 μm particle size). Methanol:water:tert-butyl methyl ether (46:42:12, v:v:v) was used as the mobile phase and the analyses were performed at 1.0 mL/min, 254 nm and 25 °C. To determine the amount of TQ, the sample was dissolved in hexane (1%), vortexed for 1 min, then 20 μL was injected into the HPLC after filtration.

Preparation of fatty acid methyl esters from olive oil method of International Olive Oil Council (IOC) (COI/T.20/Doc.24-2001)International Olive Council (IOC) (2021). Standards, Methods and Guides [online]. Website: http://www.internationaloliveoil.org/ [accessed 28 Nov 2018].  was applied and fatty acid methyl esters (FAME) compositions were determined with the test methods of transunsaturated fatty acids via capillary column gas chromatography (COI/T.20/Doc.17/Rev.1-2001) [29]. FAME analyses were performed by Agilent gas chromatography system [equipped with split/splitless (SSL) injector and FID detector] with using HP-88 capillary column (60 m length, 0.25 mm i.d and 0.2 μm film thickness). The analyses were performed with using helium as the carrier gas, the oven initial temperature was kept at 140 °C for 1 min, then increased to 240 °C with the speed of 4 °C/min and held at this temperature for 5 min. The methyl esters were formed by transesterification with cold methanolic potassium hydroxide solution. The results were expressed as relative percent peak area. The Supelco FAME Mix-37 (Bellefonte, PA, USA) standard was used to determine the location of each fatty acid methyl ester in the chromatogram.

### 2.7. Cell culture

Human normal liver epithelial cell line (THLE-2) was obtained from the American Type Culture Collection (ATCC), and was used to determine the biological activities of nanoemulsions. THLE-2 cells were chosen as a platform to study general toxicity using cell viability because of noncancerous characteristic and constitute an in vitro model for pharmacotoxicological studies.1 THLE-2 cell line was maintained in BEGM medium with modifications suggested by ATCC. Briefly, 5.0 ng/mL of EGF, 70 ng/mL of phosphoethanolamine, 10% FBS, and 100 UI/mL penicillin/streptomycin were added. Gentamycin/amphotericin (GA) and epinephrine in the BEGM Bullet Kit (Lonza) were not used in final medium. THLE-2 cells were maintained in a humidified incubator with 37 °C and 5.0% CO_2_ in air. Cells were subcultured by trypsinization twice a week.

#### 2.7.1. Cell viability

Relative cell viability was determined by colorimetric MTT assay, which based on mitochondrial oxidoreductase activity of living cells [30]. Briefly, 1 × 104 THLE-2 cells/well were incubated in 96-well cell culture plate for 72 h under standard culture conditions. After this, cells were treated with various concentrations of nanoemulsions (0.005–5 mg/mL, in BEGM) for 24, 48 and 72 h. Following this, the samples were removed completely and 110 µL/well MTT solution (0.5 mg/mL in BEGM) was added and incubated for 4 h. During this time, enzymatic activity of living cells produced insoluble formazan crystals. After the incubation, formazan crystals were dissolved in 10 % SDS (in 0.01 M HCl) (overnight) and quantified by reading the absorbance at 570 nm, and 630 nm was used as reference wavelength. BEGM without any sample was used as control and considered as 100% viable. Relative cell viability was plotted as the percent absorbance of sample treated cells (n = 5). SDS is one of the most used solvents for formazan crystals, others being DMSO and 2-propanol. The advantage of using SDS is in the final solubilisation step is that SDS can be directly added to cell medium. Thus, the risk of losing formazan crystal in the decanting of medium step is bypassed [31]. Also, with the optimum HCl amount, the solution is stable [32]. One caveat would be the remaining nonreduced MTT in the media, which can be solved by using reference wavelength of 630 nm in addition to blank readings [30].

#### 2.7.2. Cell viability after alcohol induced cell death

Relative cell viability of ethanol was determined by colorimetric MTT assay to determine the toxic dose. Briefly, THLE-2 cells were incubated in 96-well cell culture plate for 72 h under standard culture conditions. After this, cells were treated with various concentrations of ethanol (25–750 mM, in BEGM) for 24, 48 and 72 h. During 48 and 72 h incubation time, samples were renewed every 24 h to avoid alcohol evaporation [33,34]. Afterwards, the samples were removed and MTT test was performed as described previously. Experiments were performed in five replicates. According to the results, 400 mM ethanol was selected as the dose to be used.

In the second phase of the study, cell based hepatoprotective activity was performed. THLE-2 cells were grown in 96-well plates until reaching 80% confluence. Briefly, the 0.5 mg/mL nanoemulsion samples were added to wells with settled concentrations according to cell viability results and incubated for 2 h under standard culture conditions. Afterwards, samples were removed and cells were treated with 400 mM ethanol (in BEGM) for another 48 and 72 h incubation under standard culture conditions. During 48 and 72 h incubation time, samples were renewed every 24 h. At the end of the incubation process, MTT test was performed to assess cell viability, as previously described, and hepatoprotective activity of nanoemulsions against ethanol was determined. All experiments were performed in five replicates.

#### 2.7.3. Cell proliferation assay

The proliferative impact of the nanoemulsions was examined taking after the cell proliferation at various time. THLE-2 cells were treated with the 0.5 mg/mL nanoemulsion samples in 96-well plates for 2, 4, 8, 12, 24, 48 and 72 h. After these periods, samples were removed and MTT test procedure was performed as described above [35]. 

#### 2.7.4. Cell imaging via ﬂuorescence microscopy 

Nanoemulsions were loaded with fluorescein isothiocyanate (FITC) to follow nanoemulsions in THLE-2 cells by imaging with epifluorescence microscope (Olympus CKX41, CCD camera Olympus DC30, Olympus Corporation, Tokyo, Japan). Loading of nanoemulsions was achieved by adding FITC in aqueous phase of nanoemulsion preparation as described above. Samples were dialyzed to remove the surplus of FITC, which was not included in the nanoemulsion structure. 1 mg/mL nanoemulsions (in BEGM) were added to the cells grown in 6-well plate and incubated 2 h at standard culture conditions. After incubation, the cell nucleus was stained with DAPI. FITC-labeled nanoemulsions and DAPI images were merged. 

### 2.8. Statistical analysis

Results were plotted using GraphPad Prism (GraphPad Software version 5.03, San Diego, CA, USA). DPPH radical scavenging activity of A1 and A2 were compared by testing whether slopes and intercepts of linear regression curves differ significantly by using GraphPad Prism.

## 3. Results

### 3.1. Characterisation of the nanoemulsions 

The supercritical CO_2_ extraction method is among the techniques used to obtain *N. sativ*a seed oil [36]. Supercritical CO_2_ extraction method, a completely mechanical process, is superior to other methods with elimination of chemical solvent requirements and high temperature treatment. [37]. In addition, this method provides the extraction of thymoquinone derivatives, lipophilic phytochemicals and natural antioxidants without losing their biological activity [38]. Furthermore, it also allows pressure control, thus ingredients of obtained oil can be managed [36]. Therefore, *N. sativa* seed oil prepared by supercritical CO_2_ extraction method was selected for nanoformulations. The particle size, PDI and zeta potential of the BSO nanoemulsions were analysed via DLS and results are given in Table 2. According to measurements, sizes of nanoemulsion formulations are determined as 65.93 ± 12.98 nm and 65.46 ± 4.8 nm for A1 and A2, respectively. The PDI values of nanoemulsions are determined 0.485 ± 0.03 for A1 formulation and 0.47 ± 0.01 for A2 formulation. Zeta potential of A1 and A2 formulations are measured –34.57 ± 0.72 mV and –45.2 ± 0.4 mV, respectively.

**Table 2:** Particle size/zeta potential of nanoemulsions and total phenol contents of the nanoemulsions [Data represent the mean ± standard deviation (n = 3)].

Formulation	Particle size (nm)	PDI	Zeta potential (mV)	Phenolic content (µg GAE/mg sample)
A1	65.93 ± 12.98	0.485 ± 0.031	-34.57 ± 0.72	4.22 ± 0.02
A2	65.46 ± 4.80	0.472 ± 0.008	-45.20 ± 0.40	3.78 ± 0.80

### 3.2. Radical scavenging activity owing to their phenolic contents

The total phenolic content of nanoemulsions was calculated with the help of gallic acid calibration curve (1.0–500 µg/mL) as gram gallic acid equivalents per mg of nanoemulsion. Table 2 shows the polyphenol amount of the A1 and A2 nanoemulsion formulations. According to the results (Table 2), A1 formulation is found to have higher amount of polyphenols (4.22 ± 0.02) compared to A2 (3.78 ± 0.8).

**Figure 1:**
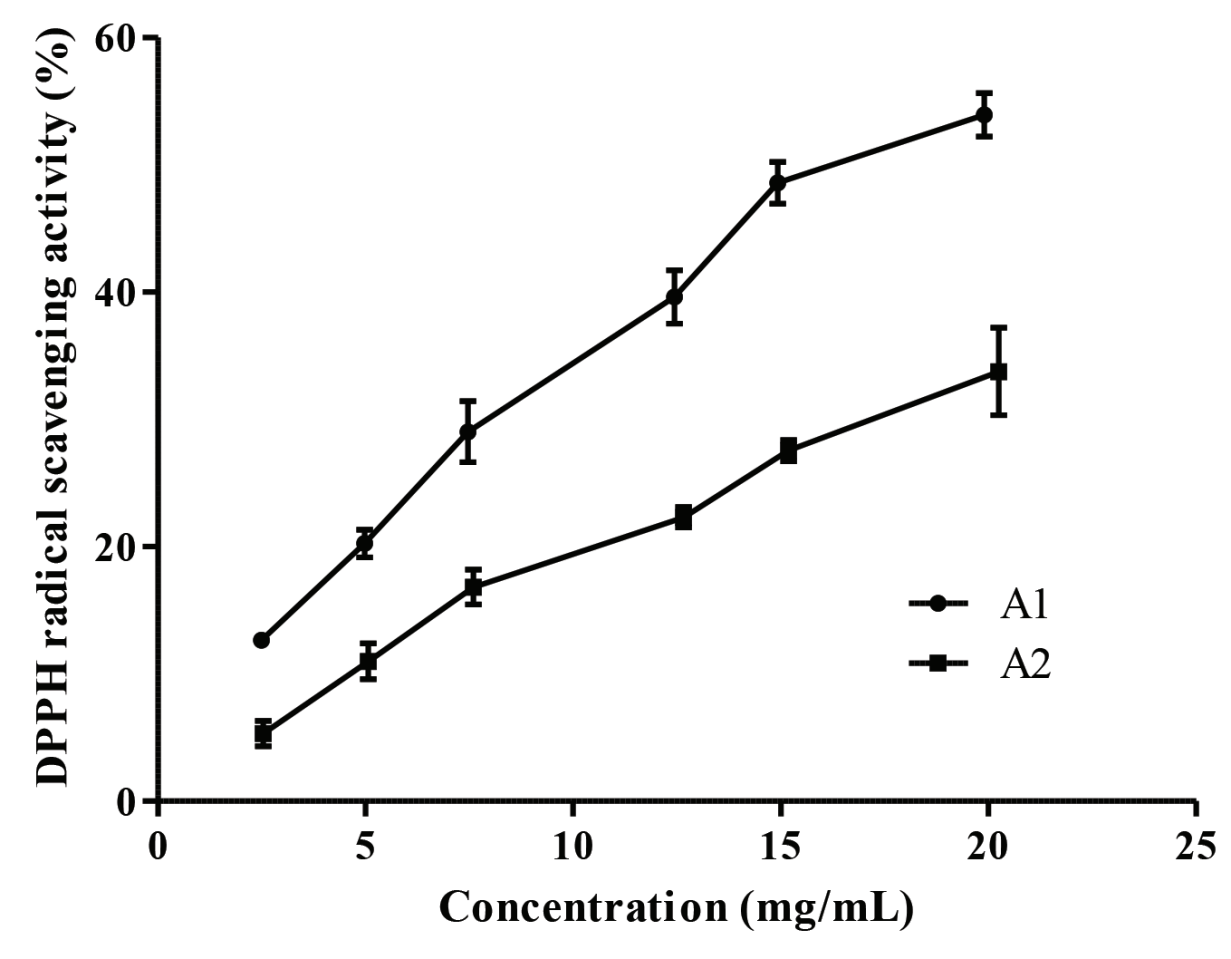
Dose-dependent radical scavenging abilities of the nanoemulsions (Bars represent the ± standard deviation (n = 3).

Free radical scavenging activity of the nanoemulsions were investigated with DPPH assays (Figure 1). A1 formulation showed significantly higher (P = 0.0048) effect on radical scavenging activity compared to A2, resulted from GSH presence in its formulation.

### 3.3. Chromatographic ingredient analyses after successful incorporation of GSH in A1, and preserved TQ and FAME ratios in both A1 and A2 emulsions

In this study, linearity, sensitivity [limit of detection (LOD) and limit of quantification (LOQ)] and accuracy (recovery) studies were performed among the validation parameters according to the International Conference on Harmonization Guidelines (ICH Q2B, validation of analytical procedures, methodology) [39]. For linearity, each calibration point was studied in 3 replicates. The LOD and LOQ were calculated according to signal/noise (S/N) ratio of 3:1 and 10:1, respectively for sensitivity. The major active ingredient of *N. sativa* seed oil is thymoquinone (TQ) [9]. Both of the nanoemulsions prepared contain black seed oil that are containing TQ. Therefore, the recovery study was calculated by blind verification for TQ. The method used for the composition of fatty acids is the standard test method of the IOC and the results are calculated on the percentage distribution by using normalization.

The calibration solutions containing GSH and TQ, which are in the concentration range of 0.1–2.0 mM and 1.0–100 (µg/mL), were obtained from the stock solutions. The calibration curve were designed to investigate the correlation between the peak area (y) and the injection concentration (x; mM and µg/mL) of GSH and TQ. Linear regression equations of the GSH and TQ were obtained at six concentration levels with triplicate injections. The linear regression equation was utilized as y = 1277.7x – 5.193 and y = 89.071x + 75.95, respectively. The linearity was expressed in terms of the correlation coefficient (R^2^). The results showed good correlation between the peak areas and the concentration of HPLC injection for GSH and TQ with R^2^ = 0.9997 and R^2^ = 0.9989, respectively.

LOD and LOQ for GSH and TQ were analysed to evaluate the sensitivity of the developed HPLC method. The LOD and LOQ for GSH were calculated 0.02 mM and 0.06 mM, respectively. The LOD and LOQ for TQ were calculated as 0.18 µg/mL and 0.62 µg/mL, respectively. 

The accuracy of the method was evaluated by determining the recovery of GSH and TQ at different concentration levels as low, medium and high level for nanoemulsion matrixes. Recovery results are given in Tables 3.

**Table 3:** Recovery results of GSH and TQ by HPLC.

Added GSH (mM)	Found GSH (mM)	Recovery (%)
0.2	0.14	70.00
0.5	0.36	72.00
1.5	1.14	76.00
Added TQ (µg/mL)	Found TQ (µg/mL)	Recovery (%)
5.0	4.53	90.60
10.0	9.62	96.20
25.0	24.42	97.68

Solvents, mobile phases and nanoemulsions solutions, which were used during analysis, were injected under same instrument conditions and any peak unwatched which had same retention time with GSH and TQ. This shows that suitability of HPLC method in terms of selectivity and there are no interferences from solvents and solution.

**Figure 2:**
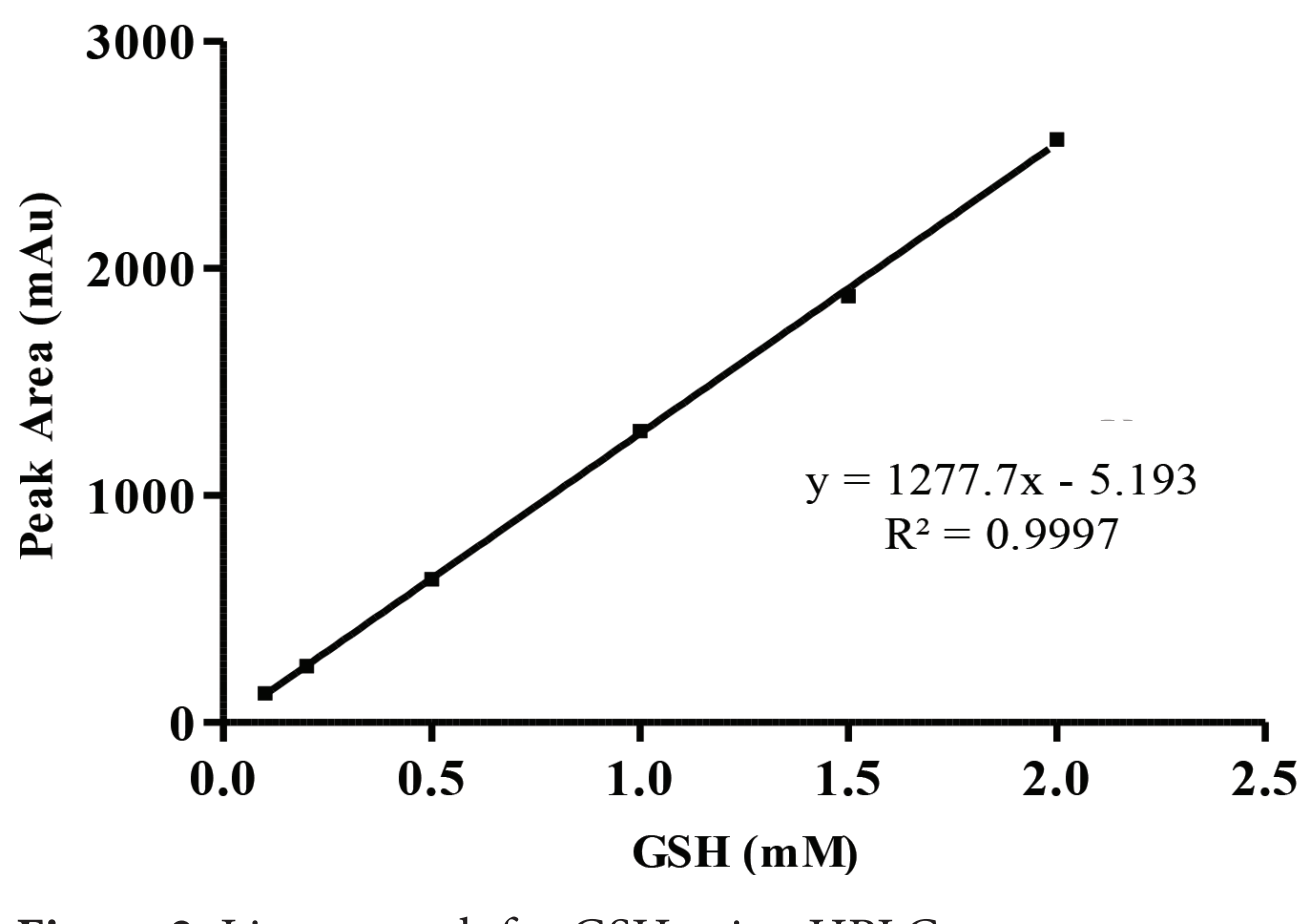
Linear graph for GSH using HPLC.

**Figure 3:**
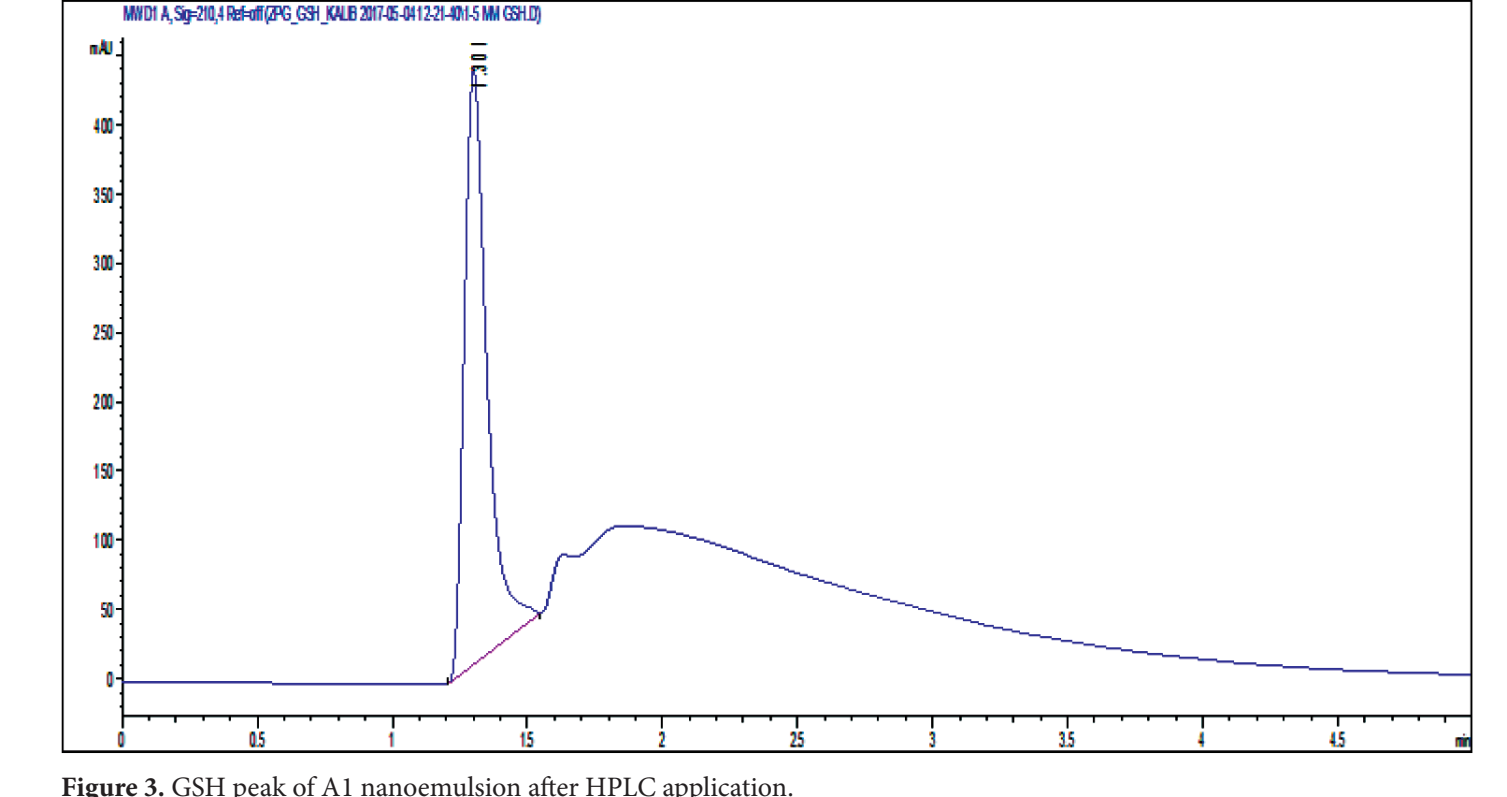
GSH peak of A1 nanoemulsion after HPLC application.

In chromatographic analysis of GSH in the prepared nanoemulsion formulations, a GSH calibration curve is drawn as the first step by HPLC (Figure 2). The amount of the GSH in the nanoemulsions is calculated with using the peak areas of GSH in the chromatograms with the help of calibration curve of GSH (Table 4). The chromatogram obtained for the A1 nanoemulsion containing GSH from these formulations is given in Figure 3. According to the results of chromatographic analyses performed to determine the amount of GSH in nanoemulsions; A1 nanoemulsion contains 0.67 mM GSH whereas no GSH is detected in A2 formulation. 

**Table 4:** The amount of the GSH present in the nanoemulsion.

Formulation	GSH (mM)
A1	0.67 ± 0.015
A2	-

**Figure 4:**
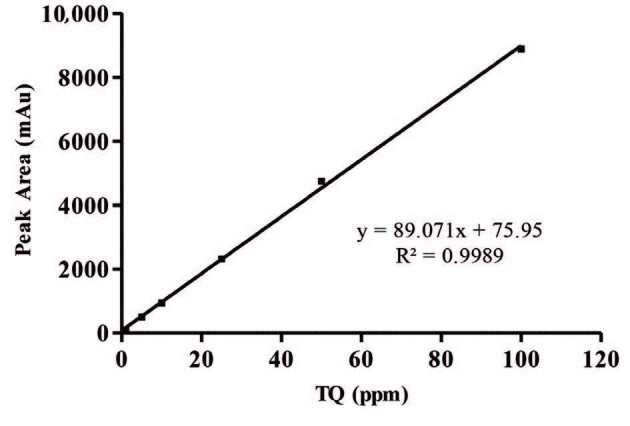
Linear graph for TQ using HPLC.

**Figure 5:**
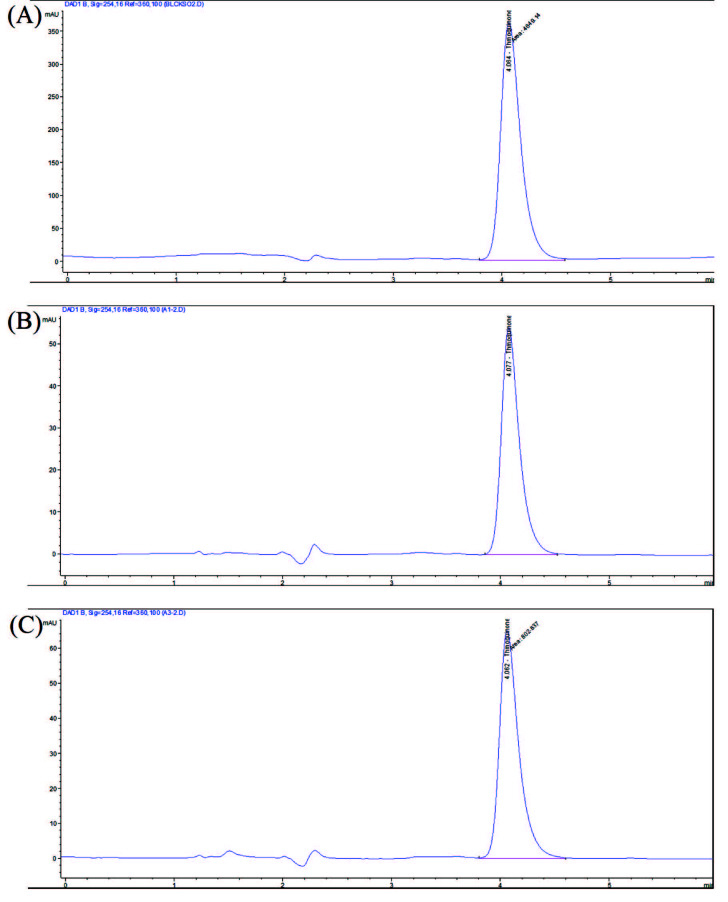
TQ chromatogram of N. sativa seed oil (A), A1 (B) and A2 (C).

The major active component of the *N. sativa* seed oil, which constitutes the oil phase of the nanoemulsions, is TQ. TQ calibration curve is drawn as the first step in HPLC analysis to determine the amount of TQ in the samples (Figure 4). The amount of the TQ present in the nanoemulsions is calculated with using the peak areas of TQ in the chromatograms by the help of calibration curve of TQ (Table 5). Chromatogram obtained for *N. sativa* seed oil is given in Figure 5A; the chromatogram obtained for the A1 nanoemulsion is shown in Figure 5B. The chromatogram obtained for A2 is given in Figure 5C. In another chromatographic analysis, which is performed to determine the amount of TQ in nanoemulsions and *N. sativa* seed oil, A1 and A2 nanoemulsions and oil contained 2622 ppm, 312 ppm and 404 ppm TQ, respectively (Table 5). The percentages of total fatty acid content as a result of GC-FID analyses of FAME in nanoemulsions and *N. sativa* seed oil are given in Table 6. The ratios in the table represent the percentage of each component in total fatty acid methyl esters.

**Table 5:** The amount of the TQ present in the nanoemulsion formulations and N. sativa seed oil.

Samples	TQ (ppm)
N. sativa seed oil	2622.59 ± 17.88
A1	312.17 ± 2.63
A2	404.40 ± 0.79

**Table 6:** The percentages of total fatty acid content as a result of GC-FID analysis of FAME in nanoemulsions and N. sativa seed oil.

FAME	N. sativa Oil (%)	A1 (%)	A2 (%)
Myristic acid (C14:0)	0.1915	0.1838	0.1925
Myristoleic acid (C14:1)	0.0432	0.0442	0.0353
Palmitic acid (C16:0)	13.0525	13.4517	13.5263
Palmitoleic acid (C16:1)	0.2190	0.3410	0.3897
Heptadecanoic acid (C17:0)	0.0726	0.0732	0.0964
Stearic acid (C18:0)	3.1782	3.6412	3.7453
Oleic acid (C18:1n9c)	24.2953	28.4358	29.7427
Linoleic acid (C18:2n6c)	55.4537	50.2815	48.6585
Arachidic acid (C20:0)	0.1880	0.2045	0.2276
Heneicosanoic acid (C21:0)	0.3491	0.3500	0.3535
Linolenic acid (C18:3n3)	0.2189	0.2163	0.2127
Behenic acid (C22:0)	2.4028	2.1957	2.1051
Cis-13,16-docasadienoic acid (C22:2)	0.0406	0.0260	0.0647
Lignoceric acid (C24:0)	0.0224	0.0198	0.0168
Cis-4,7,10,13,16,19-docasahexaenoic acid (C22:6)	0.2723	0.2838	0.2910

### 3.4. Cell viability 

**Figure 6:**
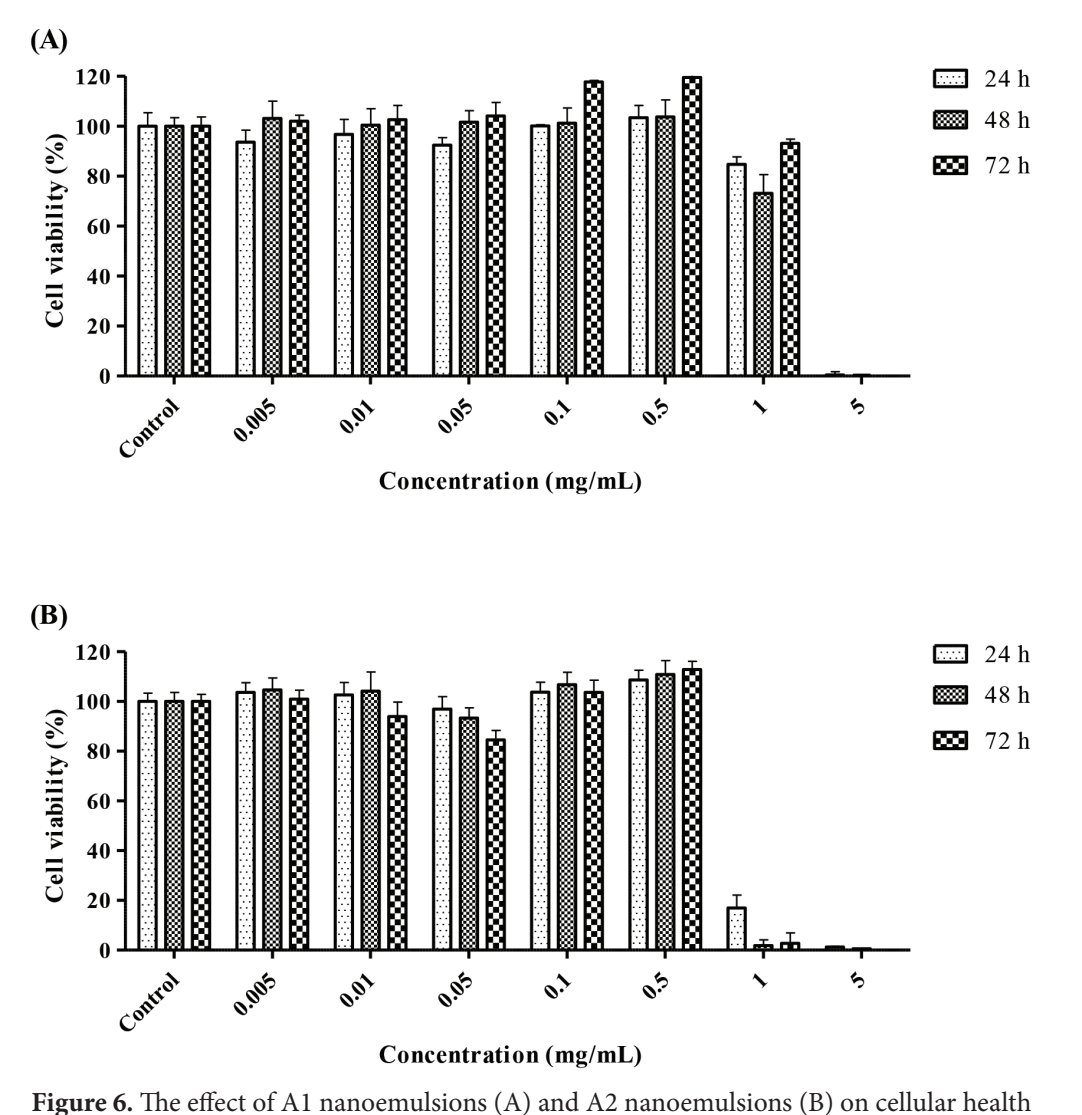
The effect of A1 nanoemulsions (A) and A2 nanoemulsions (B) on cellular health of THLE-2 cells for 24, 48 and 72 h (Bars represent standard deviation, n = 5).

THLE-2 cells were chosen to work toxicological research because this would be more appropriate in vitro model of hepatotoxicity since it is a healthy cell line and retain its normal enzymatic activities and also originated from normal human primary cells, retaining their phenotypic and functional characteristics [25,40]. In previous studies, toxicological investigations have been carried out using various carcinoma cell lines such as HepG2, C3A, HepaRG and Huh7. Even though the similar studies use hepatocellular carcinoma cell lines extensively, the rationale behind this is it being cheaper and easier to maintain. The lack of expression of main metabolizing enzymes in carcinoma cell lines limits their usability in toxicological studies [41]. Initially, experiments were performed on THLE-2 cells to determine the effect of nanoemulsions on cell viability for 24, 48 and 72 h (Figure 6). According to the results, it is seen that both A1 and A2 nanoemulsions show a negative effect on the cells at higher doses. 0.5 mg/mL of nanoemulsions, which has no toxic effect for both nanoemulsion formulations but has a positive effect on cell viability, was determined as the maximum allowable dose.

### 3.5. Protective effects of nanoemulsions against ethanol induced hepatotoxicity

**Figure 7:**
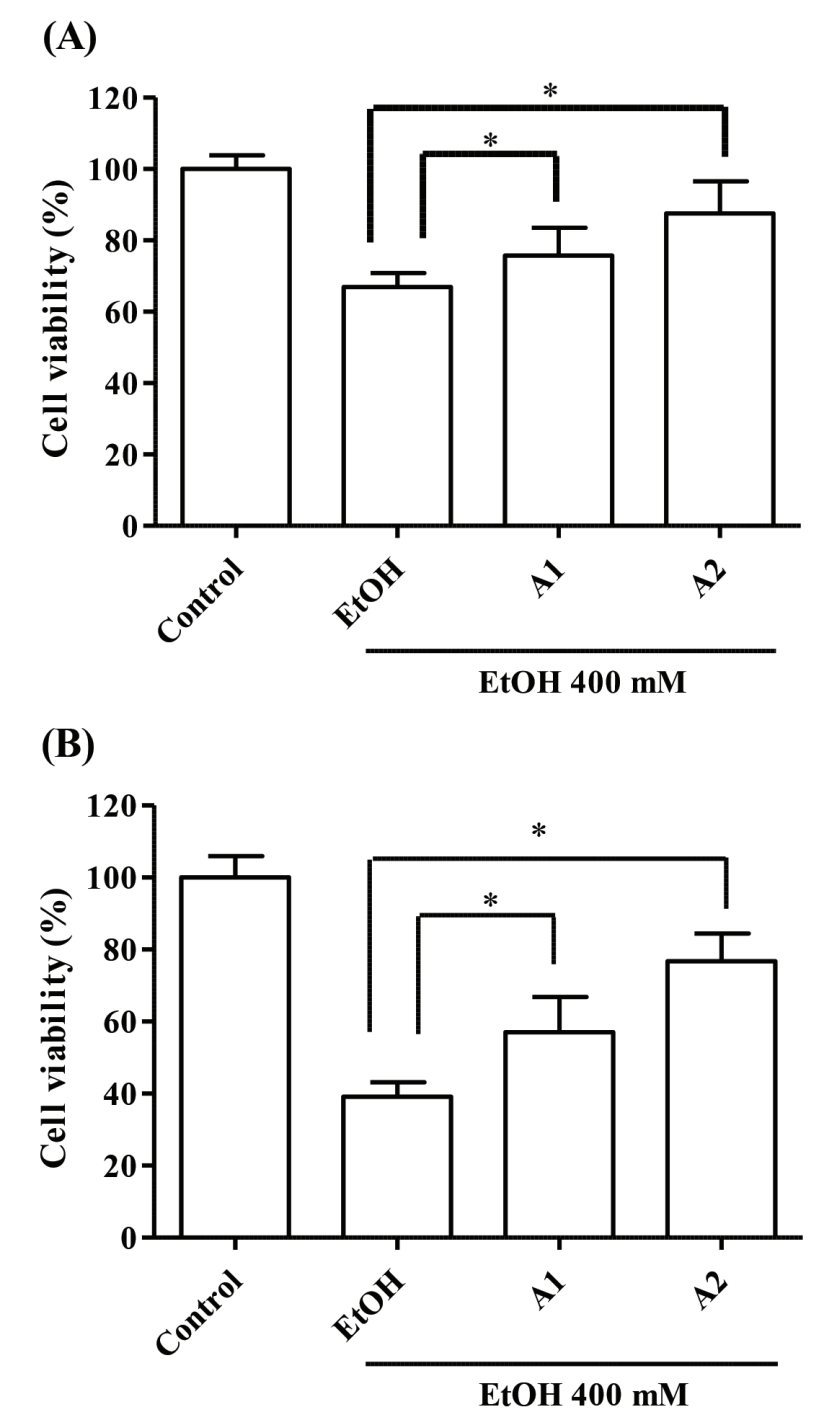
48 h (A) and 72 h (B) hepatoprotective activity of A1 (0.5 mg/mL) and A2 (0.5 mg/mL) nanoemulsions after 2 h pretreatment on THLE-2 cells (Bars represent standard deviation, n = 5). * (P < 0.05).

A model was used to assess the alterations on cell viability in the presence and absence of the nanoemulsions that were decreased by ethanol application to THLE-2 cells. Experiments were carried out by designing a model based on measuring how the damage from the application of ethanol to THLE-2 cells changed in the presence and absence of the nanoemulsions. Firstly, the concentration of ethanol to be used in the hepatoprotective activity assay was determined. And, it was decided to use 400 mM ethanol, which reduce the cell viability by approximately 50 % compared to the control group. The protective effect of nanoemulsions against damage induced by ethanol is tested for 48 and 72 h (Figures 7A and 7B). According to the results after 48 h exposure; the viability of cells pretreated with A1 is 75%, A2 is 87%, and the viability of cells not pretreated with nanoemulsions is 66%. According to the 72 h test result; the viability of cells pretreated with A1 is 57%, A2 is 76%, and the viability of cells not pretreated with nanoemulsions is 39%. 

### 3.6. Proliferative effects of nanoemulsions on THLE-2 cells

**Figure 8:**
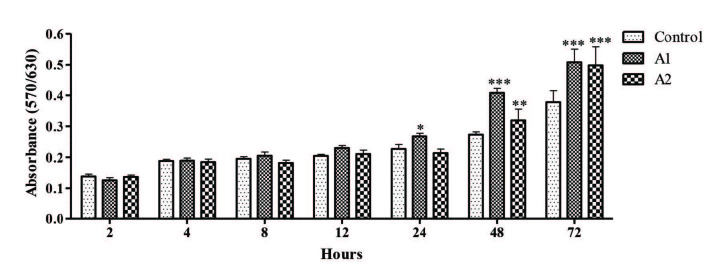
The proliferative effects of A1 (0.5 mg/mL) and A2 (0.5 mg/mL) nanoemulsions on THLE-2 cells (Bars represent standard deviation, n = 5). * (P < 0.05), ** (P < 0.01), *** (P < 0.001).

Cell viability of THLE-2 cells are obtained at designated times to determine the proliferative effects of nanoemulsions (Figure 8). According to the results, there are no significant differences after 2, 4, 8, 12 and 24 h exposure between the control group and the cells treated with A1 and A2 nanoemulsions. However, after 48 and 72 h, there are notable differences between the control group and the nanoemulsion treated cells. At the end of 48 and 72 h incubation with A1, an increase in cell viability is observed compared to the control group, 49% and 34%, respectively. A2 formulation also shows an increase in cell viability compared to the control, 17% for 48 h and 32% for 72 h.

### 3.7. Cellular localization of nanoemulsions 

**Figure 9:**
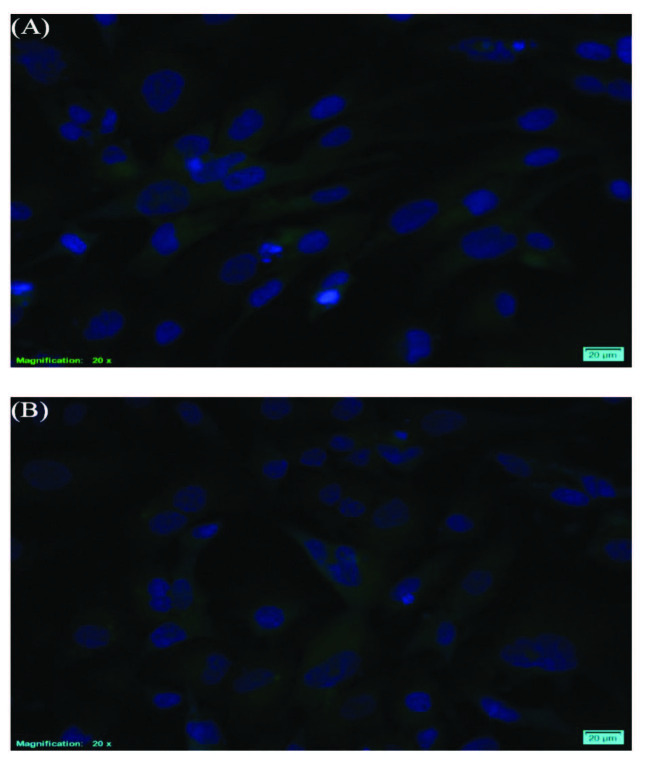
Epifluorescence images of THLE-2 cells treated with the FITC-loaded A1 (A) and A2 nanoemulsions (B). The images were obtained merging green channel (FITC) and blue channel (DAPI) images (scale bar = 20 μm).

The aim of investigating the cellular localization of nanoemulsion formulations was to demonstrate transfer ability of the nanoemulsions through THLE-2 cells membranes. Thus, the effects observed in cell culture studies are due to the penetration of nanoemulsions into the cell. Nanoemulsions were labelled with FITC, and their interactions with THLE-2 cells were monitored by fluorescence microscopy. Cells were treated with the nanoemulsions for 2 h. After that, the cell nuclei were stained with DAPI. The images were taken at the same time by using different filters in the fluorescence microscope, which are superimposed with the help of ImageJ program (shown in Figure 9).

## 4. Discussion

The nanometer-sized *N. sativa* seed oil nanoemulsions with a narrow particle size distribution were comparable to the previous works where nanoemulsions ranged between 20 and 200 nm [42], BSO nanoemulsion has nanometer-sized droplets with a narrow particle size distribution. PDI value is another factor that shows the stability of nanoemulsions, which is between 0 and 1, where former indicates monodisperse systems and latter indicates polydisperse systems [15,43]. In the light of this information, A1 and A2 nanoemulsion formulations can be considered to have relatively good homogeneity in terms of size distribution. Determination of zeta potential values is important for the stability of nanoemulsions [44]. Nanoemulsions containing highly charged surfaces were found to be more stable and more resistant to aggregation [45–47]. In this perspective, we obtained stable and uniform nanoemulsions.

Phenolics were analysed to the prove the presence of *N. sativa* seed oil in nanoemulsion formulations. According to the total phenolic content of nanoemulsions, there is difference between the two formulations. This difference may be due to the fact that the Folin–Ciocalteu reagent reacts with the thiol group-containing compounds as well as the phenolic compounds [27]. 

The free radical scavenging activities of nanoemulsions show dose-dependent efficiency. Some researchers reported that radical scavenging activity of the samples also dependent on their total phenolic content. In addition, there is a positive correlation between total phenolic content and DPPH activity [48–50]. As seen in Figure 1, free radical scavenging activity of A1 is higher than formulation A2. The reason for this difference between the two formulations is due to the antioxidant property of GSH. In addition, GSH presence in A1 nanoemulsion has been proven with chromatographic analyses. And, also chromatographic analyses of TQ ingredient in nanoemulsions; it has already anticipated that the amount of TQ will be lower than *N. sativa* seed oil due to the high water phase of the prepared nanoemulsion formulations. Besides that, the TQ content of the A1 formulation is lower than A2. This may be due to the fact that GSH and TQ can react with each other [51]. According to the chromatographic analysis, finally, it is seen that the values of the percentages of total fatty acid content measured for *N. sativa* seed oil, A1 and A2 are almost the same. 

Protective effects of nanoemulsions against ethanol induced hepatotoxicity results show that A1 and A2 nanoemulsion formulations have a high hepatoprotective effect against 400 mM ethanol on THLE-2 cell line. To the best of our knowledge, there is no alcohol related study conducted on the THLE-2 cell line, and it was observed that alcohol models were created on many different cell lines or studies were conducted on the effects of alcohol. For instance; 50 mM ethanol [52], 80 mM ethanol [53], 100 mM ethanol [34] were applied as toxic dose on HepG2 cell line; 25 mM ethanol [54] was applied as toxic dose on WIF-B cells and 10–300 mM ethanol [33] was applied as toxic dose on WRL-68 cell line. When ethanol concentration is lower than 400 mM, cell viability of THLE-2 did not be affected. All the experiments investigations were done using higher concentrations of ethanol than the ones in literature. Because of noncancerous nature of THLE-2, the presence of detoxifying enzymes in cells facilitates the regeneration of cells. Alcohol, even at lower concentrations, causes production of ROS that turns into DNA damage [24]. It can be said that 1.0 mM GSH in A1 is not enough to see better hepatoprotective activity than A2. However, there is no statistically significant difference between the two formulations on the protective effects of nanoemulsions against ethanol induced hepatotoxicity.

To further compare the A1 and A2 formulations, their effects on THLE-2 cell proliferation were evaluated. Both nanoemulsions increased proliferation of THLE-2 cells when compared to control. This effect on the cell proliferation is mainly due to oil phase of formulations, which consists of *N. sativa* seed oil [4]. Formulation A1 begun to proliferate earlier than A2 at the end of 24 h. The higher proliferation rate seen for A1 compared to A2 can be attributed to GSH in the water phase [55]. However, at the end of the 72 h, both nanoemulsion formulations showed similar proliferation rate. 

According to the cell imaging studies via fluorescence microscopy, it appears that nanoemulsions can penetrate into the cells. 

The range of the GSH concentration in cytosol is ranged between 0.1–10 mM [12,56,57]. GSH concentration is about between 1 and 2 mM in most cells. Hepatocytes carries GSH, then the level can reach about 10 mM [56]. Extracellular GSH concentrations are relatively low (e.g., 2–20 µM in plasma), except for bile acid, which may contain up to 10 mM of GSH [12]. Based on this information, we decided to use 1.0 mM GSH (the approximate level of GSH contained in a healthy living cell) in nanoemulsion formulations. In literature, Khan et al*.* prepared nanoemulsion contains paraffin oil and 450 mg of GSH in formulation [17]. When compared GSH content of previous study against to this study, GSH amount has lower A1 nanoformulations and it is enough to show its hepatoprotective effect.

## 5. Conclusion

In conclusion, nanoemulsions are important part of vesicular systems, and useful for the delivery of hydrophobic molecules in biological systems. Here, GSH enriched *N. sativa* seed oils in nanoemulsion forms were prepared, and the protective effects of these formulations against alcohol induced toxicity were tested in liver cell line. THLE-2 was used for in vitro model of hepatotoxicity research. According to the results of this study, nanoemulsion uptaken by the cells was achieved and the protective effect on alcohol induced hepatotoxicity was seen. Nanoemulsions prepared in this study, not only improve the bioavailability of nonsoluble content of *N. sativa* and but also provide GSH stability. Further research and developments in nanoemulsion preparation will be taken based on their advantages in delivery of poorly soluble drugs and phytopharmaceuticals.
